# A male presenting with a primary mucinous bladder carcinoma: a case report

**DOI:** 10.1186/1757-1626-3-49

**Published:** 2010-02-03

**Authors:** Konstantinos Sigalas, Stavros I Tyritzis, Eleni Trigka, Ioannis Katafigiotis, Nikolaos Kavantzas, Konstantinos G Stravodimos

**Affiliations:** 1 Department of Urology, Athens University Medical School-LAIKO Hospital, Athens, Greece; 2 Department of Pathology, Athens University Medical School-LAIKO Hospital, Athens, Greece

## Abstract

**Background:**

The primary mucinous adenocarcinoma of the bladder is an extremely rare urologic entity, which is found in less than 2% of all urinary bladder tumours and is often presented as metastatic.

**Case presentation:**

A 69-year old male patient was diagnosed with a primary mucinous adenocarcinoma of the bladder after undergoing a transurethral resection of a bladder tumour and complete examination of the entire gastrointestinal tract to rule out other primary cites. Immunohistochemistry confirmed the nature of the tumour. The patient underwent a radical cystoprostatectomy with en block bilateral pelvic lymphadenectomy and urinary diversion with a Bricker ileostomy.

**Conclusion:**

The primary adenocarcinoma creates a diagnostic dilemma, since it cannot be easily differentiated by the adenocarcinoma that originates from the colon and the prostate. We advocate the radical surgical management, after exclusion of any primary malignant sites related to the gastrointestinal tract. The immunohistochemistry has a leading role, assisting with the differential diagnosis.

## Background

Urinary bladder cancer is the second most frequent tumour of the genitourinary tract [1]. Adenocarcinomas account for less than 2% of all bladder cancers [2]. One of the most common forms of adenocarcinoma of the bladder is the metastatic adenocarcinoma. The primary sites for these tumours include the rectum, stomach, endometrium, breast, prostate, and ovaries. We present such a case, providing a meticulous review of the current literature.

## Case presentation

**Figure 1:**
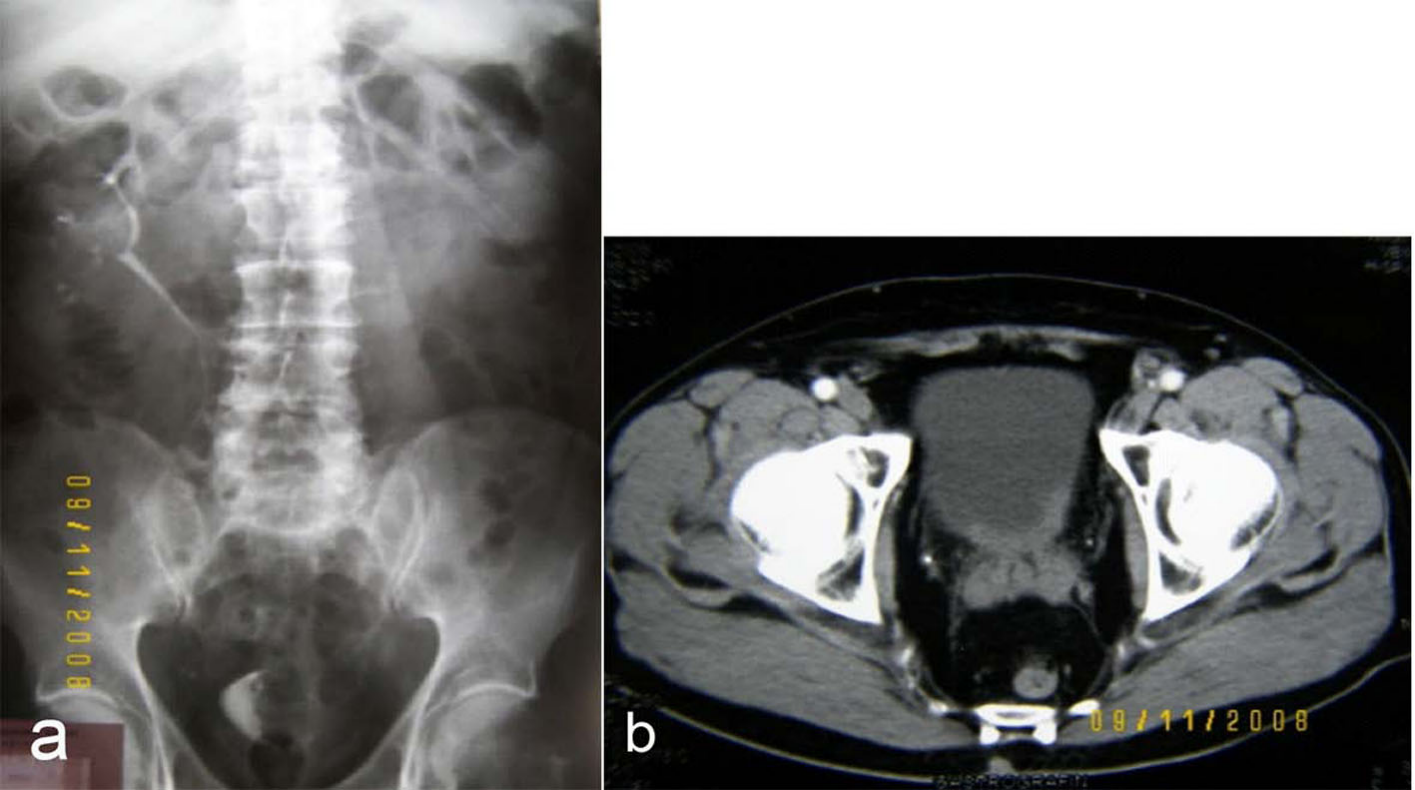
**a, Intravenous urogram showing a radiolucent filling defect in the bladder and a non functioning left kidney and b, computed tomography of the pelvis**.

A 69-year old male patient was admitted having gross painless hematuria for the last 2 months with no other comorbidities, apart from benign prostatic hyperplasia treated with a-blockers. Ultrasound of the kidneys, the bladder and the prostate showed an exophytic lesion of the bladder and dilatation of the left pelvicaliceal system. Intravenous urography (IVU) showed a radiolucent filling defect in the bladder and a non functioning left kidney (Fig. 1a). The next diagnostic step was to perform a cystoscopy, which confirmed the presence of a lesion, occupying the trigone of the bladder and the left ureteral orifice. The patient was subjected to a transurethral resection of the lesion. The histopathological assessment revealed an infiltrative mucinous adenocarcinoma. Computed tomography (CT) (Fig. 1b), colonoscopy and gastroscopy revealed no other primary malignant site. Based on the pathology report, the patient underwent a radical cystoprostatectomy with en block bilateral pelvic lymphadenectomy and urinary diversion with a Bricker ileostomy.

### Gross examination

The specimen of radical cystoprostatectomy included the urinary bladder with pericystic fatty tissue and the prostate gland. On section, a tumour was identified, measuring in the greatest dimension 3 cm. The tumour was localized in the posterior bladder wall and had an exophytic growth pattern with solid (nodular) appearance. It seemed to invade the wall of the bladder, extending to the proximal urethral margin of the prostate.

### Histological and immunohistochemical features

**Figure 2:**
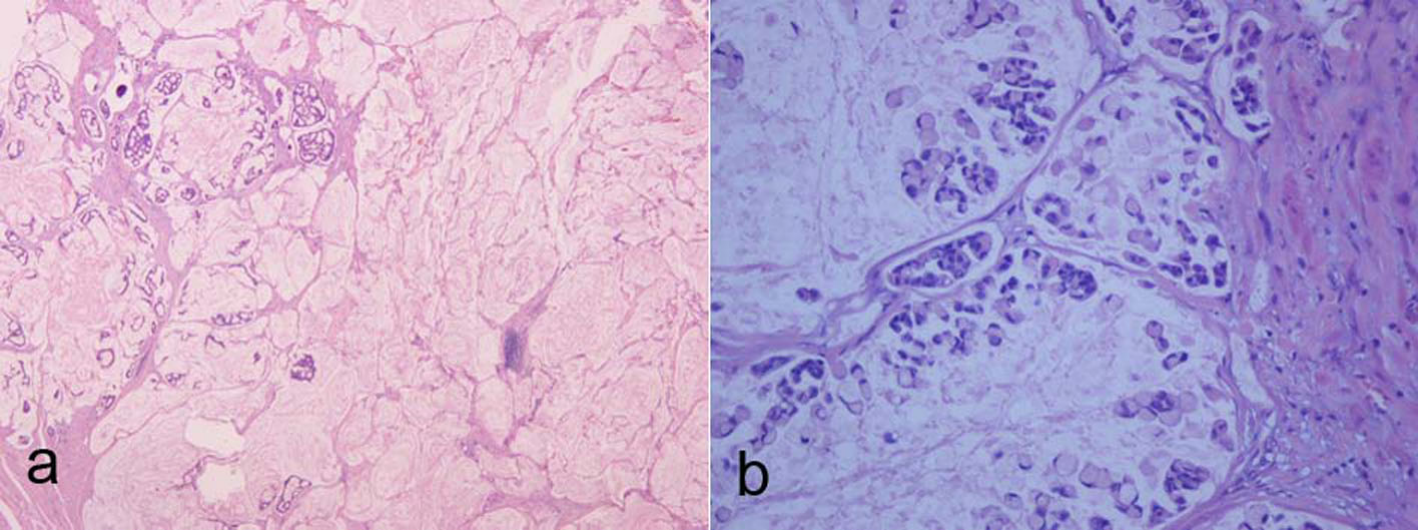
**a, Pools of extracellular mucin containing glandular configurations, b, signet-ring cells**.

**Figure 3:**
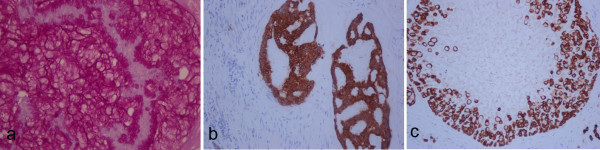
**a, Intracellular and extracellular mucin PAS-d positive, b, glandular configurations CK7 positive and c, signet-ring cells CK20 positive**.

The grossly described tumour is a primary mucinous adenocarcinoma of the urinary bladder, which invades the wall of the bladder, both lobes of the prostate gland and both seminal vesicles. We did not recognize normal urothelium with intestinal metaplasia. The carcinoma includes glandular configurations, having one cell layer of cuboidal or columnar epithelium with large, dark nuclei, signet-ring cells (Fig. 2), nuclear atypia and several mitoses (Fig. 2). The reactivity for PAS and PAS-diastase establishes the presence of intracellular and extracellular mucin (Fig. 3). The primary nature of adenocarcinoma is confirmed by the immunoreactivity for keratins 7 and 20 (Fig. 3).

## Discussion

The majority of primary adenocarcinomas of the urinary bladder (50-60%) arise at the bladder base and almost all of the remaining are associated with urachal remnants [3]. The male to female ratio of non-urachal neoplasms approaches 3 to 1, in contrast to almost 1 to 1 for urachal tumours. Most patients are middle-aged (mean, approximately 62 years). Many experts suggest that adenocarcinomas arise through a process of intestinal metaplasia stimulated by chronic irritation. Among other factors associated with urothelial adenocarcinoma, exstrophy and persistent urachal remnants are the most common. Adenocarcinomas arising in areas of urachal remnants differ clinically from those occurring at the bladder base, but these neoplasms are similar in their pathology and behavior.

Hematuria is the most common presenting sign, manifested in about 90% of patients. Almost half of the patients complain about dysuria, nocturia, frequency and pain. Cystoscopically, bladder adenocarcinomas ordinarily appear as single, nodular tumours that can not be reliably distinguished from urothelial neoplasms.

Adenocarcinomas of the urinary bladder, regardless of site, include the following histologic variations: 1) Adenocarcinoma non otherwise specified, 2) Adenocarcinoma of enteric type, 3) Adenocarcinoma with signet-ring cells, 4) Mucinous adenocarcinoma, 5) Clear cell adenocarcinoma, 6) Hepatoid adenocarcinoma, 7) Mixed adenocarcinoma [4]. The usual malignant tumour is a well-to-moderately differentiated adenocarcinoma, secreting variable amounts of mucin. The tumour cells represent a combination of columnar and goblet cells [5].

Mucinous adenocarcinoma of the urinary bladder includes large lakes of extracellular mucin mixed with collections of tumour cells. By definition, these mucinous foci should constitute at least half of the tumour mass. In some cases, there is an admixture of extracellular and intracellular mucin; the latter is resulting in signet ring configuration [6].

Regarding immunohistochemistry, adenocarcinoma of the urinary bladder expresses CEA, CDX-2, MUC-1, MUC-2 and MUC-3, same as colonic adenocarcinoma. Cytokeratins 7 and 20 are positive, in contrast with colonic adenocarcinoma that expresses cytokeratin 20 but not cytokeratin 7 [7].

The differential diagnosis includes metastatic colonic adenocarcinoma, urothelial neoplasms with glandular differentiation, intestinal metaplasia and nephrogenic metaplasia. Metastatic adenocarcinoma is differentiated using the immunophenotype (CK7 negative and CK 20 positive). Urothelial neoplasm with glandular differentiation may contain intracellular and luminal mucins; however, mucins are not abundant. In addition, in this type of carcinoma, signet-ring cells are not prominent and the "glands" are surrounded by pseudostratified epithelium. Intestinal metaplasia may infiltrate the lamina propria or even the bladder wall. Mucinous lakes are not uncommon in these cases and their presence in a tissue sample is diagnostic of adenocarcinoma only with the presence of neoplastic cells. The cells of intestinal metaplasia lack nuclear anaplasia and rarely involve the muscularis propria. Nodular areas of cystitis glandularis rich in goblet cells should be considered benign, even if the nodules extend into the lamina propria.

Prognosis varies with stage, with survival approaching 75-100% among patients whose tumours are confined to the urinary bladder. Unfortunately, low-stage cancers account for fewer than 30% of reported cases [8]. Patients with urachal tumours tend to have a better short-term survival rate than those with nonurachal cancers [9].

## Consent

Written informed consent was obtained from the patient for publication of this case report and accompanying images. A copy of the written consent is available for review by the Editor-in-Chief of this journal.

## Competing interests

The authors declare that they have no competing interests.

## Authors' contributions

KS gathered patient data. SIT gathered patient data, drafted and revised the manuscript. ET performed the immunohistochemical study and drafted the manuscript. IK drafted the manuscript and gathered reference articles. NK performed the immunohistochemical study and supervised the manuscript. KGS performed the surgical operation and supervised the manuscript.

All authors read and approved the final manuscript.
